# The Quality of Spouses’ Social Networks Contributes to Each Other’s Cardiovascular Risk

**DOI:** 10.1371/journal.pone.0071881

**Published:** 2013-08-21

**Authors:** Bert N. Uchino, Timothy W. Smith, McKenzie Carlisle, Wendy C. Birmingham, Kathleen C. Light

**Affiliations:** 1 Department of Psychology and Health Psychology Program, University of Utah, Salt Lake City, Utah, United States of America; 2 Department of Psychology, Brigham Young University, Provo, Utah, United States of America; 3 Department of Anesthesiology, University of Utah, Salt Lake City, Utah, United States of America; University of Warwick, United Kingdom

## Abstract

**Objectives:**

Although the quality of one’s own social relationships has been related to cardiovascular morbidity and mortality, whether a partner’s social network quality can similarly influence one’s cardiovascular risk is unknown. In this study we tested whether the quality of a partner’s social networks influenced one’s own ambulatory blood pressure (ABP).

**Methods:**

The quality of 94 couples’ social networks was determined using a comprehensive model of relationships that separates out social ties that are sources of positivity(supportive), negativity (aversive), and both positivity and negativity (ambivalent). We then utilized statistical models (actor-partner analyses) that allowed us to separate out the links between one’s own social network quality on ABP (actor influences), a partner’s social network quality on ABP (partner influences), and a couple’s network quality combined on ABP (actor X partner interactions).

**Results:**

Independent of one’s own relationship quality, results showed that an individual’s ABP was lower if their spouse had more supportive ties, and higher if a spouse had more aversive and ambivalent ties. In addition, couples’ networks in combination were associated with higher ABP but only if both had a low number of supportive ties, or a high number of aversive or ambivalent ties.

**Conclusions:**

These data suggest that the social ties of those we have close relationships with may influence our cardiovascular risk and opens new opportunities to capitalize on untapped social resources or to mitigate hidden sources of social strain.

## Introduction

The quality of one’s close relationships has been linked to significant health outcomes [1]–[5]. In the most compelling evidence to date, a recent meta-analysis found that positive aspects of relationships (i.e., perceived social support) were associated with a lower risk for mortality [6]. Indeed, effect sizes from the meta-analysis appeared as large, if not larger than standard risk factors such as smoking, exercise, and obesity. Of our close relationships, the quality of one’s marriage appears particularly important. It is one of the most significant adult relationships and has been similarly linked to positive health outcomes [7]–[9].

Although the evidence linking close relationships to health is relatively strong, specifying the more precise factors that contribute to such links remains an important objective to advance theory and if this work is to be used to guide interventions or health promotion efforts [10]–[11]. This study thus addresses two limitations in prior work by testing (a) if the quality of spouses’ social networks can influence their partners’ health, and (b) the more specific qualities of relationships involved in such cross-spouse associations.

Most of the prior work in this area focuses on how an individual’s relationships are linked to their own health [1]–[5]. Is it possible that the relationships of those we have close ties with might also be an important determinant of our health? Several indirect lines of research are consistent with this possibility. The important work of Christakis and colleagues [12] on “social contagion” showed that obesity was elevated in two and three degrees of social network separation. Thus, participants’ obesity levels were linked to obesity in the friends and family of their own social networks. Although little is known about the mechanisms responsible for such associations, potential possibilities include social norms and subsequent health behaviors such as eating and exercise patterns [13]. Of course, this work only examines linkages among social network ties and does not take into account the quality of the relationships.

Research in relationship science suggests that the quality of a partner’s social interactions and network ties may subsequently influence one’s own social and psychological functioning [14]–[17]. This work is consistent with interdependence theory which postulates that close relationships are characterized by a mutual dependence [18], [19]. It has been hypothesized that the quality of a partner’s relationships might influence one’s functioning in a number of ways including greater (a) affective spillover, (b) support-seeking, and (c) access to coping information [14], [17], [20]. For instance, Repetti and colleagues [21] have found strong evidence for spillover in negative social interactions at work to home interactions with a spouse. In one study, husbands and wives reported more marital anger and withdrawal at home following negative social interactions at work [17]. Other studies have found that increased contact with a spouses’ social network predicted greater positive partner processes such as viewing the spouse as a reliable source of support [14] and higher marital quality [16]. To date, however, none of this research appears to have been linked to physical health outcomes which would provide a critical bridge to epidemiological studies linking relationships to disease outcomes.

A second important issue to consider is that most of the studies on relationships and health focus on the positive aspects of relationship quality such as social support [6]. However, even relationships that are relied upon to be major sources of support are not uniformly positive and can add to a person’s distress during their time of need (e.g., feeling frustrated or let down by the support provider) [22]. This is consistent with a small epidemiological literature that has linked negativity in relationships to poorer physical health [23], [24]. Indeed, positivity and negativity in relationships are separable dimensions [22] which suggest the need for a more comprehensive approach to studying links between social ties and health. We have proposed a comprehensive framework that incorporates both of these dimensions and thus allows for an integrative approach while also elucidating a unique category of relationships that have both positive and negative aspects (i.e., ambivalent ties) [25]. Based on this framework, network members can be categorized as supportive (high on positivity, no negativity), aversive (no positivity, high on negativity), ambivalent, or indifferent (no positivity or negativity).

In the current study, we tested whether the quality of a spouse’s social relationships beyond the marriage can influence one’s ambulatory blood pressure (ABP) study during daily life. We focused on ABP as an outcome because it is an important predictor of future cardiovascular risk even when considering clinic blood pressure levels [26]. To separate out the links between one’s own relationships and a spouse we utilized actor-partner models [27]. These models allowed us to test if a person’s social network quality predicts their own ABP (i.e., actor influences). More importantly, it allows a test of whether a partner’s social network quality predicts one’s own ABP (i.e., partner influences), as well as if the couple’s combined social network quality predicts their own ABP (i.e., actor X partner influences). Consistent with prior work focusing on one’s own social relationships, we predicted that one’s supportive ties would predict lower ABP whereas one’s aversive and ambivalent ties would be related to higher ABP (actor hypotheses). We further predicted that one’s ABP would be lower if their spouse had more supportive ties, and heightened if their spouse had more aversive or ambivalent ties (partner hypotheses). Finally, we examined if the combination of both partner’s network quality was linked to ABP. We predicted that one’s own and a partner’s supportive ties in combination would be associated with lower ABP, whereas the combination of one’s own and a partner’s aversive and ambivalent ties would be linked to higher ABP (actor X partner hypotheses). In general, indifferent ties were not predicted to be linked to ABP given their limited influence [25], [28].

## Methods

### Participants

The study was approved by the Institutional Review Board at the University of Utah (IRB 00028220). Written informed consent was obtained from 97 healthy couples who were recruited through advertisements placed in local newspapers, workplace newsletters, and flyers distributed around the community. We used the following criteria to select healthy participants based on our prior work: no existing hypertension, no cardiovascular prescription medication use, no history of chronic disease with a cardiovascular component (e.g., diabetes), and no recent history of psychological disorder such as major depressive disorder [29]. Participants were all legally married and living together with a mean age of 29.6. Most participants were White (83%), college educated (62.4%), and had an income over $40,000 per year (66%). Three couples who did not follow the study protocol were eliminated from the study, resulting in a total of 94 couples. Participants were compensated $75 or received extra course credit for their time.

### Procedures

Eligible participants first arrived at the laboratory on the morning of a typical work day as part of a larger program project. Height and weight were assessed using a Health-o-Meter scale in order to calculate body mass index to be used as a covariate. Demographic (e.g., age, income, and education) and health information (e.g., smoking) were collected and participants completed the Social Relationship Index [28]. Participants then underwent a one day ABP assessment, typically from 8 am to 10 pm (*M* = 14.01 hours, *SD* = 0.97) which included working hours and an evening at home with the spouse. The ABP monitor was set to take a random reading once within every 30 minute window. This random interval-contingent monitoring procedure minimizes participants’ anticipation of a blood pressure assessment that might lead them to alter their activities. Following each ABP assessment, individuals were instructed to complete questions that assessed basic control variables such as posture and activity level which were programmed into a palm pilot device. Participants were instructed to complete these questions within 5 minutes of cuff inflation. Participants were fitted with the ABP monitor by a trained research assistant and given detailed instructions on how to use it, including how to remove it at the end of the day. One reading was obtained before the participants left the lab to insure that the monitors were working properly and that participants understood how to use the palm pilots. Participants were compensated and debriefed at their final return appointment.

### Assessments

#### Social Relationships Index (SRI)

The SRI instructs individuals to list the initials of individuals in the following domains: (a) father, (b) mother, (c) other family, (d) friends, (e) co-workers, and (f) social acquaintances. The categories of other family, friends, co-workers, and social acquaintances are limited to 5 people in order to keep completion of the SRI to a manageable time frame. These network members are then rated in terms of how helpful and upsetting they are (1 = not at all, 6 = extremely) when the participant needs emotional, tangible, and informational support. These positivity and negativity questions load on distinct factors and have relatively high test-retest reliability [28]. Based on our prior work, we operationalized different categories of social relationships as the total number of individuals in one’s network who were sources of indifference (i.e., “1” on both positivity and negativity), support (i.e., “2” or greater on positivity and only a “1” on negativity), aversion (i.e., only a “1” on positivity and “2” or greater on negativity), or ambivalence (i.e., “2” or greater on both positivity and negativity). Although the SRI can be used to assess marital quality, the primary social network quality analyses reported here do not include this relationship in order to focus on social network influences beyond the marriage.

#### Ambulatory blood pressure

The Oscar 2 (Suntech Medical Instruments, Raleigh, NC) was used to estimate ambulatory systolic blood pressure (SBP) and diastolic blood pressure (DBP). The Oscar was developed to meet the reliability and validity standards of the British Hypertension Society Protocol [30]. The cuff was worn under the participants’ clothing, and only a small control box (approximately 5.0×3.5×1.5 inches) attached to the participant’s belt was partially exposed. Outliers associated with artifactual readings were identified using standard criteria by Marler, Jacobs, Lehoczky, and Shapiro [31]. These included: (a) SBP<70 mmHg or >250 mmHg, (b) DBP<45 mmHg or >150 mmHg, and (c) SBP/DBP< [1.065+ (.00125 X DBP)] or >3.0. Readings were taken once randomly during each 30 minute window.

#### Ambulatory Diary Record (ADR)

Participants were instructed to complete a series of programmed questions following each ambulatory cardiovascular assessment using the Purdue Momentary Assessment Tool [32]. The ADR was designed to be easy to complete (about 2–3 minutes) in order to maximize cooperation. It contained information on basic variables that might influence ABP [33]. These included posture (lying down, sitting, standing), activity level (1 = no activity, 4 = strenuous activity), location (work, home, other), talking (no, yes), temperature (too cold, comfortable, too hot), prior exercise (no, yes), and prior consumption of nicotine, caffeine, alcohol or a meal (no, yes). Readings were examined to ensure compliance and discarded if not instigated within 5 minutes of a blood pressure reading. The average participant had less than one reading dropped from analysis due to noncompliance (*M* = 0.78).

#### Health assessment

A standardized health questionnaire provided information on the following potential health-related variables: weekly exercise habits, use of tobacco products (no, yes), weekly alcohol consumption, and body mass index (calculated from height and weight that was directly measured with a health-o-meter scale). The health behavior questionnaire has been used in a large longitudinal study on the chronic stress of caregiving for a relative with Alzheimer’s Disease and its effects on physiological function [34].

### Data Analysis

We utilized PROC MIXED (SAS institute) in order to examine actor-partner network quality influences on ABP [35]–[36]. PROC MIXED uses a random regression model to derive parameter estimates both within and across individuals [37]. All factors were treated as fixed [38] and PROC MIXED treats the unexplained variation within individuals as a random factor. In the present study, we modeled the covariance structure for the two repeated measures factors of dyad (i.e., husband, wife) and measurement occasion (i.e., reading number). Such nested repeated measures designs can be handled in PROC MIXED by specifying separate covariance structures for each of the factors [39]–[40]. Based on the recommendations of Park and Lee [40], we modeled the covariance matrices for dyad and measurement occasion using the “type = un@ar(1)” option. The Satterthwaite approximation was used to determine the appropriate degrees of freedom [36].

The resulting actor-partner models allowed one to test if one’s own network quality (actor influences) and a partner’s network quality were significantly ((*p*<.05) related on one’s outcomes [27]. Preliminary analyses showed that age, gender, household income, body mass, posture, temperature, activity level, prior alcohol, and prior exercise were independent predictors of higher ambulatory SBP (p’s<.05). In addition, age, gender, household income, body mass, posture, activity level, and a prior meal independently predicted ambulatory DBP (p’s<.05). Consistent with prior work, these factors along with time (i.e., first reading, second reading) were statistically controlled in all analyses involving ABP [33]. We then tested these actor-partner models by examining each social network category separately (i.e., supportive, aversive, ambivalent, indifferent) and its links to ABP with all variables centered at the grand mean. These models included both actor and partner social network variables so that each predictor was independent of the other. Finally, we tested actor X partner interactions by including the centered actor and partner main effects followed by the actor-partner cross product term [27].

## Results

### Descriptive Results

We first examined the prevalence of different social network categories in our sample. As might be expected, most social network members were supportive (*m* = 8.39, *sd* = 4.48). Importantly, a relatively large proportion of network members were also sources of both positivity and negativity (i.e., ambivalent, *m* = 7.92, *sd* = 4.26). The number of aversive (*m* = 1.02, *sd* = 1.38) and indifferent (*m* = 0.77, *sd* = 1.48) ties were predictably less prevalent. These proportions are consistent with our prior work [28]. The different network types were also only moderately correlated with each other. The number of supportive ties was negatively related to the number of aversive (*r* = −.18) and ambivalent ties (*r* = −.46) and not related to the number of indifferent ties (*r* = −.06). The number of ambivalent ties was also negatively related to the number of indifferent ties (*r* = −.24) but not aversive ties (*r* = −.06). Finally, the number of aversive and indifferent ties were positively related to each other (*r* = .35).

### Does One’s Own Social Network Quality Predict ABP (Actor Influences)?

Consistent with prior work, the quality of one’s own networks was related to health outcomes. As predicted, the extent of one’s own supportive ties was inversely related to ambulatory DBP (*b* = −.16, *SE* = .06, *p* = .01). In addition, the number of aversive ties was related to higher ambulatory SBP (*b* = 1.75, *SE* = .39, *p*<.001) and DBP (*b* = 1.11, *SE* = .20, *p*<.001), whereas the number of ambivalent ties was marginally related to higher ambulatory SBP (*b* = .18, *SE* = .10, *p* = .07). The number of indifferent ties did not predict ABP (*p*’s>.32). It is important to note that unlike prior work, these models take into account partner influences and thus test the unique influence of a person’s own social network quality on their health outcomes.

### Does a Partner’s Social Network Quality Predict One’s Own ABP (Partner Influences)?

Results also revealed support for our hypotheses regarding the role of a partner’s social networks on one’s ABP. The degree of support in a partner’s network was related to lower levels of one’s own ambulatory SBP (*b* = −.18, *SE* = .10, *p* = .055). Moreover, the number of aversive partner ties was related to higher levels of ambulatory SBP (*b* = .82, *SE* = .30, *p* = .006) and DBP (*b* = .38, *SE* = .20, *p* = .05). In regards to ambivalent ties, participants whose partners had more of such ties in their network had elevated ambulatory SBP (*b* = .27, *SE* = .10, *p* = .01) and DBP (*b* = .13, *SE* = .06, *p* = .04). No partner influences were evident for the number of indifferent ties (*p*’s>.41). Thus, even after taking into account one’s own social networks quality, partner social network characteristics uniquely predicted a person’s ABP.

### Does a Couple’s Social Network Quality in Combination Predict ABP (Actor X Partner Influences)?

**Figure 1:**
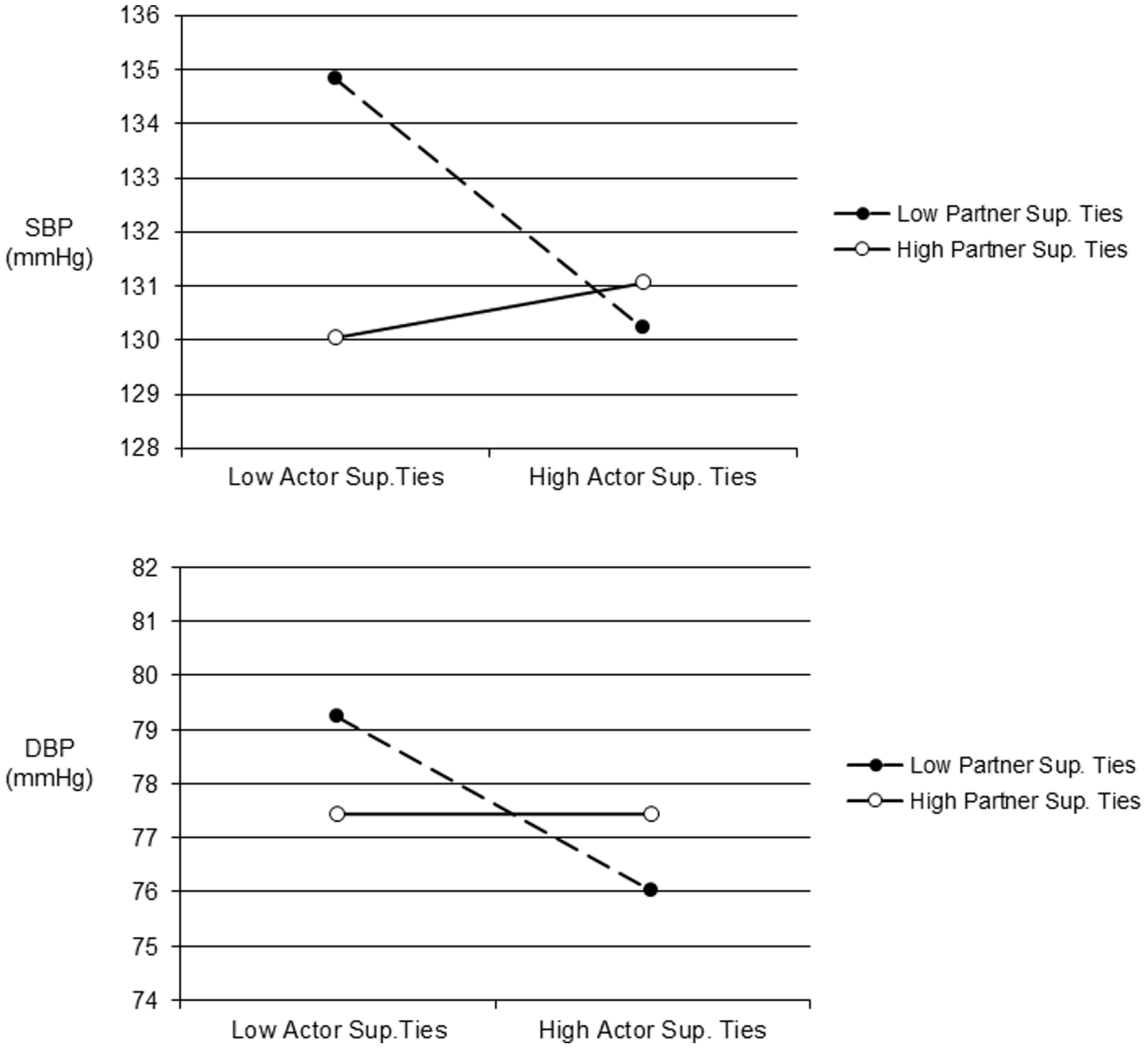
Predicted ambulatory SBP (top panel) and DBP (bottom panel) one standard deviation above and below the mean for the number of actor and partner supportive ties. Note: Sup. = Supportive.

Finally, consistent evidence was found that considering a couple’s social networks together predicted one’s ABP. Actor-partner interactions were evident for supportive ties on ambulatory SBP (*b* = .07, *SE* = .02, *p* = .01) and DBP (*b* = .04, *SE* = .02, *p* = .01). We examined the form of these interactions by plotting predicted values one standard deviation above and below the mean for actor and partner supportive ties [41]. As shown in Figure 1, ABP was highest only when both an individual’s own supportive ties and their partner’s supportive ties were low.

**Figure 2:**
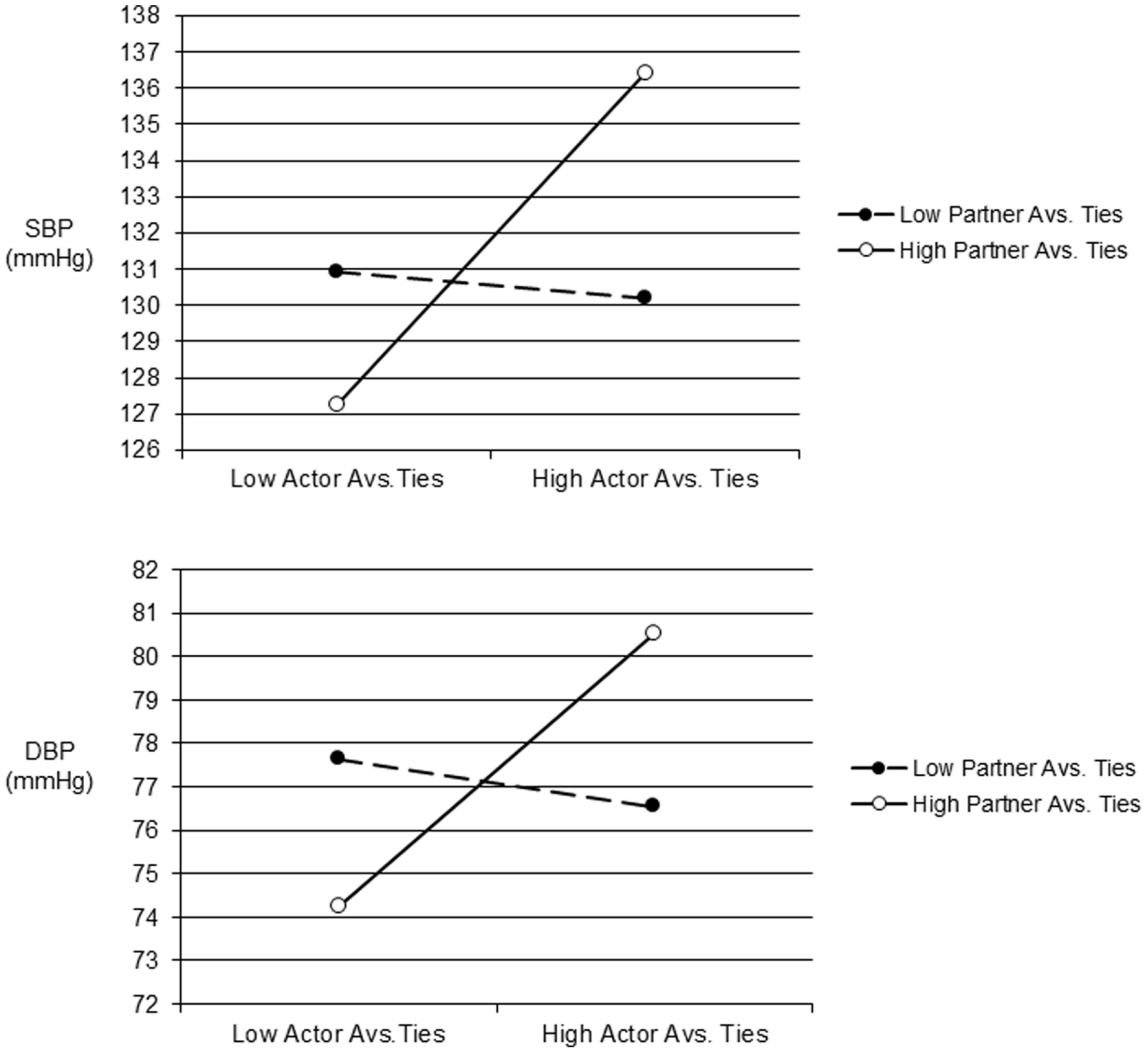
Predicted ambulatory SBP (top panel) and DBP (bottom panel) one standard deviation above and below the mean for the number of actor and partner aversive ties. Note: Avs. = Aversive.

**Figure 3:**
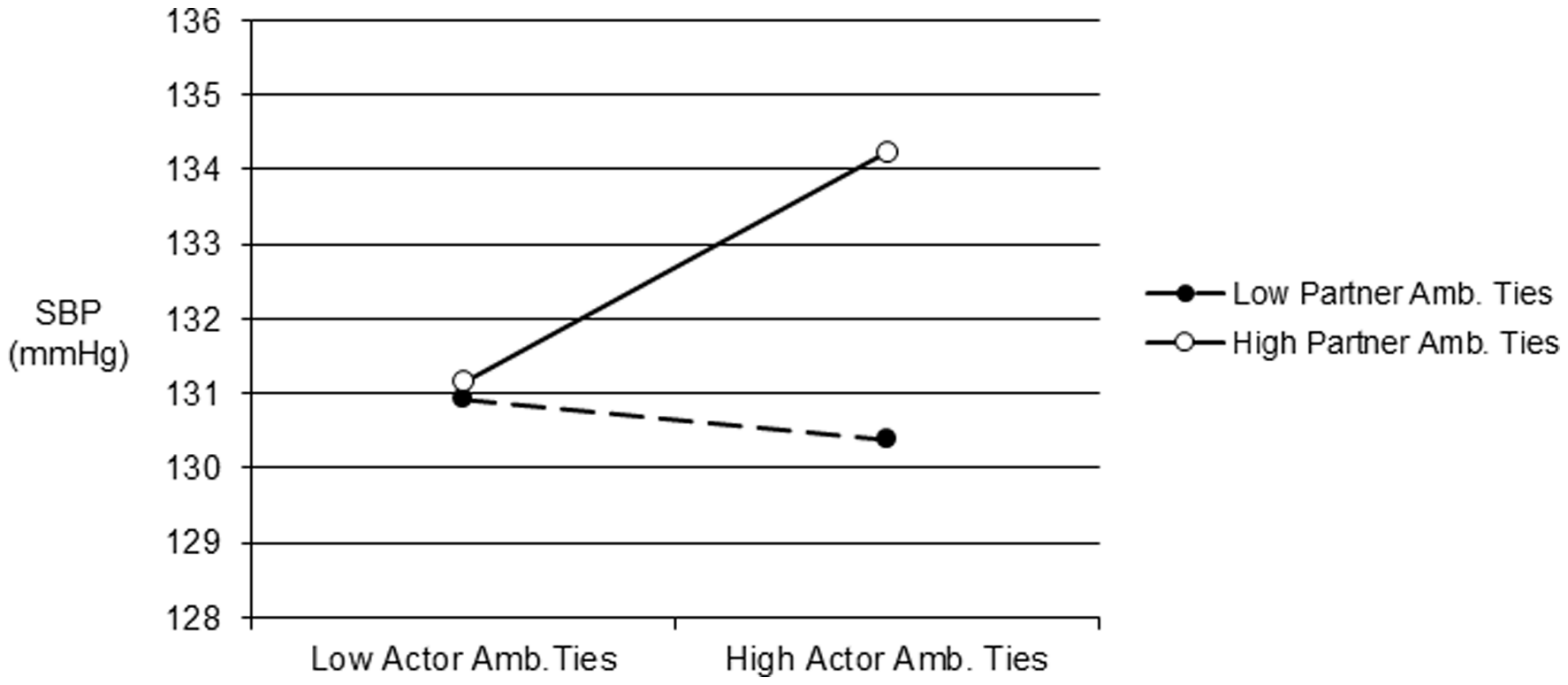
Predicted ambulatory SBP one standard deviation above and below the mean for the number of actor and partner ambivalent ties. Note: Amb. = Ambivalent.

We also found actor X partner interactions for aversive ties on ambulatory SBP (*b* = 1.30, *SE* = .20, *p*<.001) and DBP (*b* = .97, *SE* = .13, *p*<.001). We again plotted predicted values as detailed above and found that ABP was elevated primarily when a participant and their partner both had more aversive ties (see Figure 2). Consistent with our prior work indicating negative influences of ambivalent ties, a significant actor X partner interaction on ambulatory SBP also showed that SBP was highest primarily when participants and their partners both had more ambivalent relationships (*b* = .05, *SE* = .03, *p* = .04, see Figure 3).

Finally, although not predicted we also found an actor X partner interaction for the number of indifferent ties on ambulatory SBP (*b* = .48, *SE* = .18, *p* = .01). For this interaction, ABP was primarily elevated only when both participants’ and their partners’ social networks were relatively high in indifferent ties. Of course, given these results were not predicted and was the only significant link for indifferent ties appropriate caution is necessary in interpreting this isolated result.

### Exploratory Analyses

We also conducted ancillary analyses aimed at examining if these results were relatively independent of each other. We considered these results exploratory given the increased complexity of the models (i.e., actor, partner, and actor X partner cross-product scores for ambivalent, supportive, aversive, and indifferent ties) in this moderate sample size [42]. The only associations that changed appreciably were the partner support main effect on SBP (p = .57) and the actor X partner interactions for ambivalent and indifferent ties on SBP (p’s <.34). Thus, for most major findings the same pattern of results emerged in these more conservative analyses.

## Discussion

Although the quality of one’s own social relationships has been related to health outcomes [1]–[6], very little is known about the contribution of a spouse’s social relationships to such links. The main goal of this study was thus to examine if a spouse’s social network quality was related to a person’s ABP using an integrative model of relationships that considered both positive and negative aspects. Besides replicating prior work on the health benefits of one’s own social relationships, we found consistent evidence that a spouse’s relationships also influence one’s own cardiovascular risk. Indeed, the predicted values for couples low in support, or high in aversive or ambivalent ties (see Figures 1–3) meet or exceed the cut-off for normal ABP that corresponds to disease risk for SBP [43].

The main findings from this study were that a partner’s social networks were linked to the ABP of their spouses. Being married to a spouse who had less supportive ties was associated with higher levels of ABP, whereas being married to a spouse who had more aversive or ambivalent ties was related to higher levels of ABP. It is important to note that such partner influences were modeled such that they were independent of one’s own supportive, aversive, or ambivalent ties. We also found that the combination of one’s own and a partner’s relationships was related to ABP. Higher ABP was primarily evident if participants and their spouses both had less supportive ties, more aversive ties, and more ambivalent ties. These findings are consistent with work in relationship science suggesting that individuals in close relationships are mutually dependent on each other and such processes can influence marital interactions [18]–[19]. However, this is the first study that we are aware of that directly links this dependence to one’s own disease risk.

The results of this study might be viewed as consistent with social contagion influences [12]–[13]. Research on social contagion suggests that obesity can spread through related social networks up to three degrees of social network separation and might be influenced by health behaviors. Of course, such studies only consider the linkages among social networks, unlike the present study which take into account the quality of the relationship. However, it does raise the issue of whether our results are due to health behaviors and/or obesity. Inconsistent with such mechanisms, our models statistically controlled for body mass index and analyses also found that statistically controlling for smoking status, weekly exercise frequency, and weekly alcohol consumption resulted in the same pattern of results for this study. Thus, these links do not appear to be due to differences in health behaviors which in turn influence ABP.

So what are the potential processes by which a partner’s social relationships can influence a person’s ABP? Although future research will be needed, one possibility is related to increased affective spillover as negative interactions outside the home can carry over to home interactions and increase feelings of anger towards the spouse [17]. Aversive and ambivalent relationships in a partner’s social network may also trigger defensive anger due to concerns regarding the impact such relationships have on a partner’s well-being. Regardless of its source, this is relevant because anger in marriage has been linked to greater coronary calcification [44]. The link between a partner’s supportive ties and one’s own health might be due to lower access to support and coping options as the availability of supportive social network members can be a rich source of informational and emotional support which might in turn help individuals understand, accept, or cope more effectively with their own sources of stress [5], [14]. Alternatively, the lack of supportive ties may deplete an individual by leading one to expend more personal resources when coping with stress [45].

There are several limitations of this study that should be noted. First, all individuals were healthy so whether these findings result in clinically-relevant cardiovascular changes over time need further study. ABP, however, is a strong continuous predictor of future cardiovascular risk and the predicted values for individuals low in support, or high in aversive or ambivalent ties meet or exceed the risk cut-off for normal ABP [43], [46]. Second, it is possible that a couple’s social networks overlap somewhat with one another [16]. The extent of this issue cannot be determined in this study as only minimal identifiable information was obtained from the SRI. Nevertheless, prior work suggests this overlap is modest at best [16] and even if there were some overlap our statistical models separated out how couples’ perceived the quality of these relationships. Future research using more detailed social network information and complex modeling can address this issue, especially to test if these two facets (i.e., structure, quality) interact to potentiate any possible links.

## Conclusions

Given the personal and economic burden of cardiovascular disease, it becomes of utmost importance to identify modifiable risk factors that can be targeted for intervention [47]. This study extends prior work by identifying a partner’s social relationships as influences on one’s own cardiovascular health. This work highlights the interdependence inherent in close relationships while also identifying untapped coping resources and hidden sources of strain that can be targeted for intervention via couples’ therapy or cognitive behavioral interventions [48]–[49].
